# Some General and Special Facts Concerning Syphilis

**Published:** 1883-10-20

**Authors:** 


### SOME GENERAL AND SPECIAL FACTS CONCERNING SYPHILIS.

Rarely within the first year, and generally after, tertiary syphilis arises. The symptoms and signs of this stage are: Skin eruptions of severe types; bone diseases; diseases of the viscera. Of the skin diseases rupia, ecthyma and tubercular disease are common. These three are easily distinguished from each other. Rupia, at first a papule, then a vesical, then an ulcer covered by a small greenish scab. The ulcer does not heal, but under the crust it enlarges and thus creates (the result of exposure to the air, etc.,) another crust under the first and slightly larger than it. The process continues until the patient presents numerous green pyramidal crusts, covering underlying ulcers. The formation of the rupial crust can be easily explained, thus: Let a dime represent the first crust; place under the dime a larger coin, a penny; under this a five-cent piece; under this a silver quarter, and the stratified pyramidal crust of rupia is explained.

Ecthyma begins like rupia, but instead of a round or circular shape is oval. It is covered by a yellowish green oyster shell-like flattened crust which, as in rupia, covers an ulcer.

Tubercular eruptions consist of nodules deposited in the dermal and subdermal tissues. These nodules are the seat of pain of a constant dull but severe nature. Their tendency is to ulcerate, and when this occurs they may become covered with crusts or remain ulcers, generally with sharply cut edges. Rupia, ecthyma and tubercular eruptions leave cicatrices. They occasion more or less constitutional disturbance. They have their favorite sites upon which to locate. Rupia and ecthyma appear most commonly on the thighs and legs; the back, arms, forearms, etc. Tubercular syphilis appears anywhere, principally on the face, forearms, legs, etc. All three prefer the outer portions of the limbs.

Gummatous tumors under the skin now and then appear. The regions abounding in lax connective tissue are their favorite seats; thus, they are met with on the scrotum, etc. Dermal gummata are rather rare. "Nodes" or bone "gummata," are tumors under the periosetum separating it from the bone. Here they occasion severe pain, continuous, aggravated at night-time. The pain in gummata of the skin is slight, as a rule. Bone diseases, caries and necrosis, often occur in late syphilis, and generally the result of the formation of gummata. These gummatous tumors are the result of inflammation, localized, in which the inflammatory product effused gradually assumes a gelatinous consistence and is composed of cells resembling those of granulation tissue. The tumor forms under the periosteum, lifting it from the bone and destroying the blood channels by stretching or otherwise preventing nutrition of the surface of the bone, hence death of the surface or caries results. Inflammation of the whole bone may occur, resulting in the formation of gummata within the cancellous structure and so interfering by inflammatory exudations with the circulation of the bone that necrosis results. Caries is often, necrosis rarely met with. A curious result of syphilitic ostitis is hardening or ivory-like condensation of inflammatory effusions.

A patient in the Philadelphia hospital died of pneumonia. He had been a syphilitic; had had ostitis of the tibiæ; section of the right tibia showed diffuse sclerosis of the bone. The head ot the tibia was enlarged, and in its center were ivory-like condensations of bone; the same condition was seen in the left tibia, but to a less extent. Gummata most frequently occur on the sternum, the tibiæ, the cranium; but any bone may be thus attacked. I have known a syphilitic gumma of a metacarpal bone laid open under the impression that subperiosteal abscess existed.

Internal syphilitic disease (sometimes called quarternary syphilis) means disease of the viscera. Syphilitic subjects frequently show, on post-mortem, large cicatrices in the liver, most probably the former seat of gumma, which has undergone fibroid change, or having been absorbed, was succeeded by a growth of fibroid tissue. Of the spleen the same may be said. The lungs are liable to a deposit of small gummata or tubercle which, when well developed constitutes a form of "consumption" which, under treatment, gives good results. All tertiary syphilitic lesions are the result of some form of inflammation. The lungs, liver, spleen, testicles, and brain are the viscera most commonly affected; any organ may, however, become the seat of disease, and the diseases which are most common are gummata, fibroid degenerations, amyloid degenerations, growths of connective tissue, and other degenerations, as in blood-vessels the early occurrence of atheroma points to syphilis. A kidney disease, amyloid degeneration, is now and then met with and exhibits all the symptoms of that trouble. At the same time it must be remembered that a better prognosis may be given for those cases of amyloid disease when occasioned by syphilis than when due to other causes. The virus of syphilis acts only when sufficiently concentrated and, also, the quality ot the virus undergoes with age a change.

Proofs: First. The virus of syphilis requires time after the primary lesion to produce secondary symptoms as eruptions, iritis, etc. This is called a period of incubation; fermentation, growth, or concentration of the virus, expressed more plainly. When an outbreak occurs the virus is for the time being exhausted. Why? The skin, endeavoring to eliminate from the supercharged blood, the poison accompanies its assumed function to a certain degree; when, however, this enumctory is thus severely taxed for a variable period, the poison occasions inflammatory skin troubles, as papules, etc. Now comes a stage of rest, absence from eruptions, etc.; the virus is multiplying, growing, and when again sufficiently concentrated another outbreak occurs. Second. The virus changes its character. 1. Take, for instance, the appearance of the lesion in secondary syphilis and contrast them with the serious lesions of tertiary syphilis. One and the same virus, without changing its quality or character, would produce the same lesions, but the lesions of tertiary syphilis differ greatly from those of primary and secondary syphilis. 2d. The auto-inoculability of primary lesions is very low: the auto-inoculability of tertiary lesions (pus from ecthyma, etc.,) is greater and will produce lesions allied to those from which they are taken. 3rd. Primary and some secondary lesions will produce when inoculated from, upon a non-syphilitic, chancre followed by general syphilis. The virus from a tertiary lesion will rarely produce primary syphilis when inoculated upon a non-syphilitic. 4th. The child of a syphilitic parent or parents, before tertiary syphilis has become manifest, will most likely be the subject of inherited syphilis, presenting generally secondary symptoms within a few months of its birth. The child of parents long the subject of tertiary lesions rarely develops syphilitic conditions, commonly they are strumous. From these considerations I think it is justifiable to infer that the virus of syphilis is modified by age, and, also, that a certain quantity of the virus is requisite before it can or does produce syphilitic signs or symptoms.

The treatment of tertiary syphilis is simple, and as a rule, satisfactory. An important point is the nutrition of the patient. The best of food, plain but substantial and plenty of it, brandy two to four or six ounces per day, will frequently alone cause the simple and less complex symptoms to disappear; iodide of potassium is here as valuable as it is worthless in primary and early secondary syphilis.

The dose must depend upon circumstances. Mercury salts should also be given to counteract active virus, but it must be watched and given in small quantities. Rupia, ecthyma and ulcerated tubercles may be advantageously treated by removing the scabs and crusts and applying an iodoform ointment of iodoform with a mercurial ointment—as a rule this treatment does not leave such marked scars as if the crusts were left alone. Internal treatment must, of course, be given at the same time. When ulcerated tubercles are very obstinate the application every third day of one part to seven of the acid nitrate of mercury in addition to the above frequently facilitates cicatrization. If the ulcer be deep, obstinate and large, the application of a lead plate (see November number, 1882, on "Treatment of Ulcers by Lead Plate") will cure the case. Tonics, fresh air, cleanliness, good food, are invaluable. For visceral lesion, the iodides with mercury; and in a lung case especially, the iodide of ammonium, is of great service. Nervous diseases due to syphilis are treated in the same manner, care being exercised to keep the muscles from undergoing degeneration by the current, farradic or galvanic.

Gummatous tumors, wherever situated, should never be opened unless they threaten the destruction of the patient. Large doses of the iodides remove them.—*Western Med. Rep.*

